# First-principles study of the structure of water layers on flat and stepped Pb electrodes

**DOI:** 10.3762/bjnano.7.47

**Published:** 2016-04-11

**Authors:** Xiaohang Lin, Ferdinand Evers, Axel Groß

**Affiliations:** 1 Institut für Theoretische Chemie, Universität Ulm, 89069 Ulm, Germany; 2 Institut für Theoretische Physik, Universität Regensburg, 93040 Regensburg, Germany

**Keywords:** density functional theory calculations, Pb surfaces, stepped surfaces, vibrational spectrum, water structure

## Abstract

On the basis of perodic density functional theory (DFT) calculations, we have addressed the geometric structures and electronic properties of water layers on flat and stepped Pb surfaces. In contrast to late d-band metals, on Pb(111) the energy minimum structure does not correspond to an ice-like hexagonal arrangement at a coverage of 2/3, but rather to a distorted structure at a coverage of 1 due to the larger lattice constant of Pb. At stepped Pb surfaces, the water layers are pinned at the step edge and form a complex network consisting of rectangles, pentagons and hexagons. The thermal stability of the water layers has been studied by using ab initio molecular dynamics simulations (AIMD) at a temperature of 140 K. Whereas the water layer on Pb(111) is already unstable at this temperature, the water layers on Pb(100), Pb(311), Pb(511) and Pb(711) exhibit a higher stability because of stronger water–water interactions. The vibrational spectra of the water layers at the stepped surfaces show a characteristic splitting into three modes in the O–H stretch region.

## Introduction

The interaction of water with metals is of immense technological importance as it is relevant in, e.g., electrocatalysis, electrochemical energy conversion and storage, and corrosion. At the same time this interaction is of fundamental interest as there are still many open questions left with respect to the structure of liquid–solid interfaces and the influence of the liquid on properties of the metal [1]. The importance of understanding the electrochemical behavior of electrode and electrolyte near the interfaces is well illustrated by two recent examples.

(i) In recent experiments on molecular break junctions it was found that certain molecules (methyl-sulfide-bearing thiophenes) exhibit a rectification ratio two orders of magnitude larger than it has ever been observed before in single (or few)-molecule junctions [2]. This technological breakthrough is due to the experiment being performed in an electrochemical environment, namely a polar solvent (propylene carbonate). The phenomenon has not been understood well so far. As an explanation it was proposed that the solvent would enhance asymmetries in the voltage drop, which could originate from different atomic configurations of the source/drain contacts [3]. (ii) As is well-known, the electrode potential can also be used to control structural properties. This is related, e.g., to the fact that the surface free energy, and therefore also the surface-induced strain, is sensitive to the structure of the Helmholtz layer. In this spirit, electrochemical structure control has recently be successfully employed to realize a single-atom switch by reversibly manipulating atomic-scale quantum point contacts in an electrochemical environment resulting in a single-atom transistor [4–7] that exhibits an outstanding stability at room temperature. This opens attractive perspectives to prepare quantum devices based on atomic-sized conductors [8–12]. However, the microscopic mechanisms underlying the operation of the single-atom transistor [4] are not clear in detail yet.

In order to contribute to the understanding of the electrochemical single-atom switch, we addressed fundamental properties of electrode/electrolyte interfaces based on first-principles electronic structure calculations. As Pb has been used as one of the metallic electrode materials, we have already studied the Pb self-diffusion on flat and stepped Pb surfaces [13] as this controls the growth mechanism of the contacts. The results presented in this work have been done in the framework of the doctoral thesis of Xiaohang Lin [14], and first preliminary results with respect to water structures on flat Pb surfaces were recently reported [15]. Furthermore, structural and vibrational properties of water on stepped metal surfaces at finite temperatures were addressed using ab initio molecular dynamics (AIMD) simulations [16] yielding good agreement with the experiment [17], and the influence of the presence of ions in aqueous electrolytes on the transport properties of atomic junctions was assessed [18] taking into account an appropriate coverage of adsorbed ions on the junction [19–21].

As part of this ongoing research programme, here we present a detailed computational study on the structural and vibrational properties of water layers on flat and stepped Pb surfaces. Besides its relevance for the understanding of microscopic details of the electrochemical single-atom switch, this study also yields interesting insights into the structure of metal/water interfaces in general. Because of their fundamental importance, metal/water interfaces have been studied quite extensively [1,22–25], also from a theoretical point of view [26–33]. Most of the studies have focused on the structure of water on late transition metals because of their importance in electrocatalysis, Usually, it had been assumed that water forms crystalline ice-like single layers on closed-packed (111) metal surfaces because of the matching hexagonal geometry [22–23,29,34–38]. However, Pb has a much larger lattice constant than typical d-band metals, which is in fact too large to allow for the formation of a hexagonal hydrogen-bonded network [15]. Note that the particular structure of adsorbed water layers results as a consequence of the balance between water–water and water–metal interactions which are of comparable strength [15,29,36,39]. Furthermore, AIMD simulations revealed that at finite temperatures even on the close-packed (111) d-band metal surfaces the ice-like structure is not longer stable, but rather becomes disordered [30,40].

In the present work, we have addressed structural and electronic properties of water layers on flat and stepped Pb surfaces using periodic density functional theory (DFT) calculations. We will show the consequences of the large lattice constant of Pb on the resulting structure of the adsorbed water layers. We have performed AIMD simulations at a temperature of 140 K to address thermal effects in the stability of the water layers. Furthermore, thus we could also derive vibrational spectra of the water layers which will be compared to those on other metal surfaces.

## Theoretical Methods

Periodic DFT calculations have been performed employing the Vienna ab initio simulation package (VASP) [41–42] within the generalized gradient approximation (GGA) to describe the exchange–correlation effects, using the Perdew, Burke and Ernzerhof (PBE) exchange–correlation functional [43]. This functional has been chosen to allow for a better comparison with our previous studies addressing Pb surfaces [13,15]. The PBE functional is known to reproduce metal properties well, also structural water properties are described satisfactorily [44–45]. Still, PBE leads to an over-structuring of water [44–45]. Furthermore, PBE does not yield the correct wetting behavior of water on metals [32]. Taking into account dispersion effects in the water–water and water–metal interactions remedies these deficiencies [32–33,46–47]. Still it has been found that the relative stability, adsorption sites, and adsorption geometries of competing water adstructures are relatively insensitive to the inclusion of dispersion effects [33]. Nevertheless, we have tested the effect of taking into account dispersion effects by using the dispersion-corrected RPBE-D3 functional [48–49] which leads to a reliable description of both water–water and water–metal interactions [32,46,50]. The binding energies per water molecule of water structures on Pb(111) with coverages of 1/3 and 2/3 are increased by less than 70 meV upon including dispersion, in particular, the energetic ordering is not changed, as found before [32–33]. Note that the RPBE-D2 scheme [51] has recently been applied to model molecular interactions with supported Pt–Pb hybrid nanoparticles [52].

The one-particle states were expanded in a basis of plane waves up to an cutoff energy of 400 eV. The surfaces have been modeled by slabs of a certain thickness. For the low-index Pb(111) and Pb(100) surfaces, a thickness of five layers turned out to be sufficient to obtain convergent results. For example, water adsorption energies varied by less than 10 meV when low-index Pb slabs with 5, 7, 9 and 11 layers were considered. The fact that the properties of low-index Pb slabs are basically converged for a thickness of five layers has also been found in a previous DFT study [53]. For Pb(311), Pb(511) and Pb(711) 10, 15, and 20 layers, respectively, were necessary. However, note that for example the Pb(711) layer has (100) terraces that are four atomic rows wide so that a Pb(711) slab with 20 layers corresponds to (100) terraces that are five layers thick. Hence, in principle the same effective layer thickness has been used for low-index and high-index Pb surfaces. The vacuum region in our model is set to 20 Å. The Pb lattice constant was derived from our calculation for bulk Pb yielding 5.02 Å, whose accuracy is acceptable compared to the experimental value of 4.95 Å.

In order to obtain minimum-energy configurations, structures were relaxed until the residual forces were smaller than 0.01 eV/Å within a 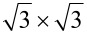 supercell for Pb(111), a 2 × 2 supercell for Pb(100) and 1 × 3 supercells for stepped Pb surfaces. A *k*-point sampling of 5 × 5 × 1 *k*-points was used to perform the integration over the first Brillouin zone. AIMD simulations were performed within the microcanonical ensemble using the Verlet algorithm with a time step of 1 fs at a temperature of 140 K, starting with the optimized structures and performing the statistical averages after thermalization of the water layer. Vibrational spectra of the water layers were derived from the Fourier transform of the velocity-velocity auto-correlation function [30].

The adsorption energy per water molecule is calculated according to

[1]
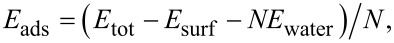


where *E*_tot_, *E*_surf_ and *E*_water_ correspond to the energies of the metal–water system, the bare surfaces and the isolated water molecule, respectively, and *N* is the number of water molecules per supercell. The energy of the isolated water molecules has been determined using a single water molecule in a supercell of size 20 Å × 20 Å × 20 Å. This energy does also not change when we use an asymmetric unit cell of size 20 Å × 20.1 Å × 20.2 Å in order to avoid artifacts due to symmetry.

As upon adsorption water forms a hydrogen-bonded network, *E*_ads_ includes both the water–metal and the water–water interaction. In order to determine the “pure” water–water interaction, the isolated water layer within the same geometry as the adsorbed layer has been considered. The strength of the water–water interaction can then be derived from

[2]
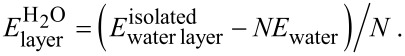


However, note that upon adsorption the water–water interaction is modified because of the water–metal interaction, and there is no unambiguous way of disentangling both contributions to the water adsorption energy [29,36].

## Results and Discussion

**Figure 1:**
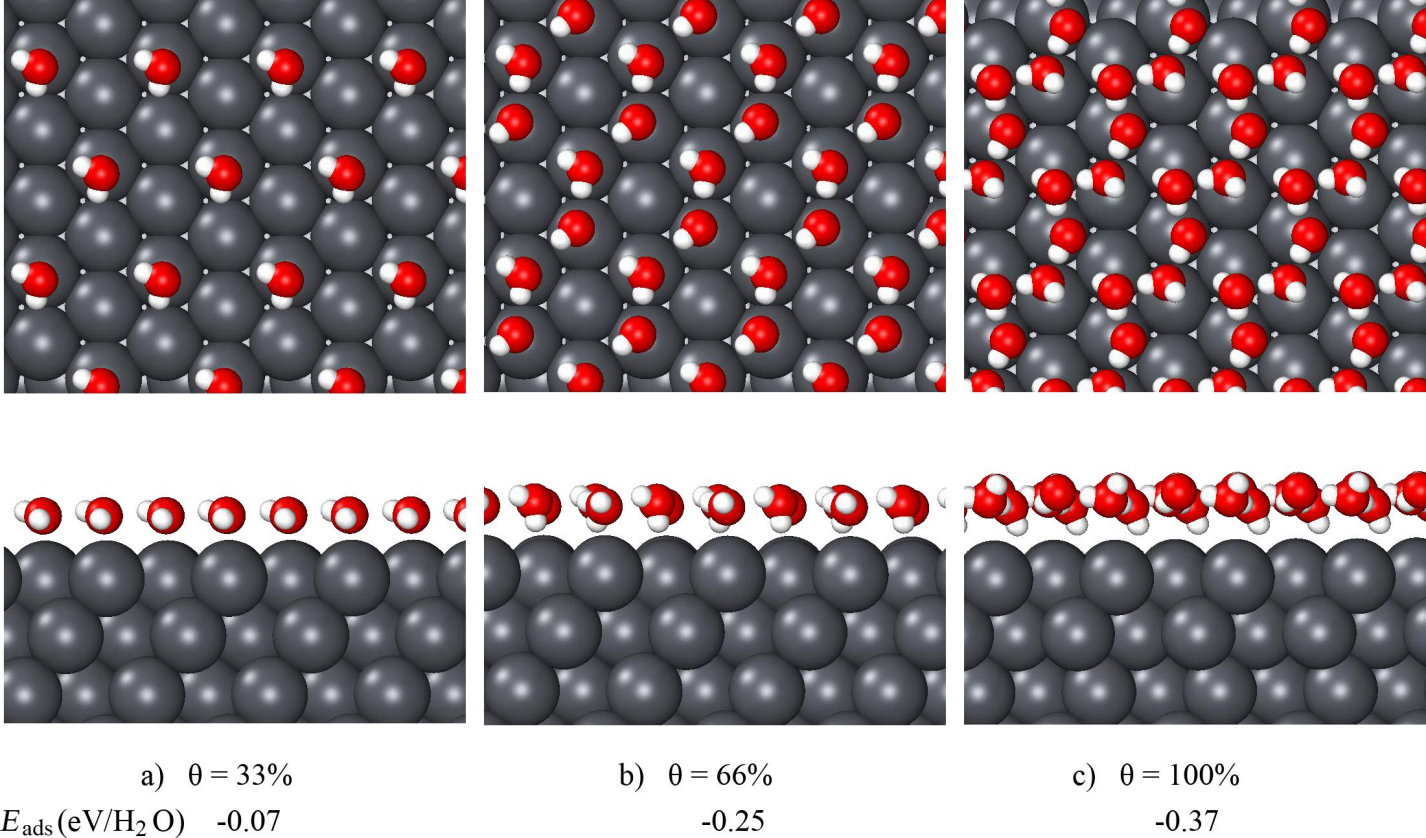
Top and side views of water structures on Pb(111) at different coverages, θ. The Pb, O and H atoms are colored in grey, red and white, respectively. The adsorption energies are shown at the bottom of the panels. In panel a, the periodic images of the water molecules have been suppressed.

As a first step, we consider the adsorption of water on the low-index (111) and (100) surfaces. A single water molecule binds to Pb(111) in the usual fashion [29] through its oxygen atom (see Figure 1a), however, with the relatively small adsorption energy of −0.07 eV at a coverage of 1/3. Reducing the coverage to 1/9 changes the adsorption energy by less than 10 meV. This adsorption energy is even smaller than the one of a water monomer on Ag(111) or Au(111) [29]. This indicates that the water–Pb interaction is rather weak.

The hexagonal ice-like structure typically corresponds to the minimum-energy structure for one water layer on a close-packed (111) surface. Figure 1b shows the optimized ice-like water structure on Pb(111). It is obvious that the water layer does not form closed hexagons, but rather a stripe-like structure. As the side view demonstrates, the water layer is rather flat. The distance between the oxygen atoms of the water molecules and the metal surface is about 3.7 Å. The distances between the oxygen atoms differ to a certain extent. The shortest one is about 3.01 Å, but the average distance is 3.5 Å. In general, the adsorbed water molecules are not hydrogen-bonded to three other water molecules, but just to two. This results in a rather low adsorption energy of −0.25 eV, which is only almost one half of the value on Ag(111).

In order to better understand the reasons for the low adsorption energy of the ice-like layer on Pb(111), we try to decompose the water adsorption energy into water–water and water–metal interactions. Such decompositions have been carried out before [29,36,54], however, note that there is no unambiguous way to decompose these contributions as water–water and water–metal interactions influence each other [29,36], which can be understood considering simple bond-order considerations [55–56]. In Table 1 the adsorption energies of ice-like layers on Pb(111) and Ag(111), *E*_ads_, are compared to the binding energies of the free-standing water layers in the corresponding adsorption geometry, 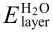. First of all, it is obvious that the adsorption of the ice-like layer is much stronger on Ag(111) than on Pb(111). Also the binding energy 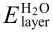 of the free-standing water layers in the Ag(111) adsorption geometry is much larger than in the Pb(111) adsorption geometry. The differences between *E*_ads_ and 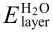 for the Pb and Ag geometries are rather similar. This is a strong indication that it is the enlarged distance of the water structures on Pb(111) compared to Ag(111) that causes the weak adsorption of ice-like rings on Pb(111). In other words, at a coverage of 2/3 the water–water attraction is reduced on Pb(111) compared to Ag(111) because of the lattice constant of Pb, which is 21% larger than the one of Ag.

**Table 1:** Calculated adsorption energies of water layers at a coverage of 2/3 on Pb(111) and Ag(111) compared to the binding energies of free-standing water layers in the corresponding adsorption geometries.

	*E*_ads_ (eV)	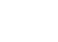 (eV)	lattice constant (Å)

Pb(111)	−0.254	−0.221	5.02
Ag(111)	−0.450	−0.395	4.09
difference	0.196	0.174	21%

This large lattice constant of Pb leads to a surface unit cell the area of which is 46% larger than that of Ag. Consequently, a water layer with a coverage of 1 on Pb(111) has almost the same density of adsorbed water molecules per area as a water layer with a coverage of 2/3 on Ag(111). And indeed, we find a rather stable water layer with coverage 1 on Pb with an adsorption energy of −0.37 eV, its structure is illustrated in Figure 1c. The oxygen atoms are located almost in one plane, and there are three different kinds of water molecules in the supercell in an H-up, H-down and parallel configuration. The water molecules are no longer all located above the top sites of the surface. The distance between the oxygen and the Pb substrate atoms is 0.6 Å larger than for the 2/3 coverage structure, indicating an even weaker Pb–water interaction, which is, however, overcompensated by the stronger water–water interaction because of the formation of the hydrogen-bonded water network. Still, there is no clear structural motif associated with this particular water structure on Pb(111). It might be regarded as a strongly distorted octagon. Hence it should also be interesting to consider water structures on the square Pb(100) surface.

**Figure 2:**
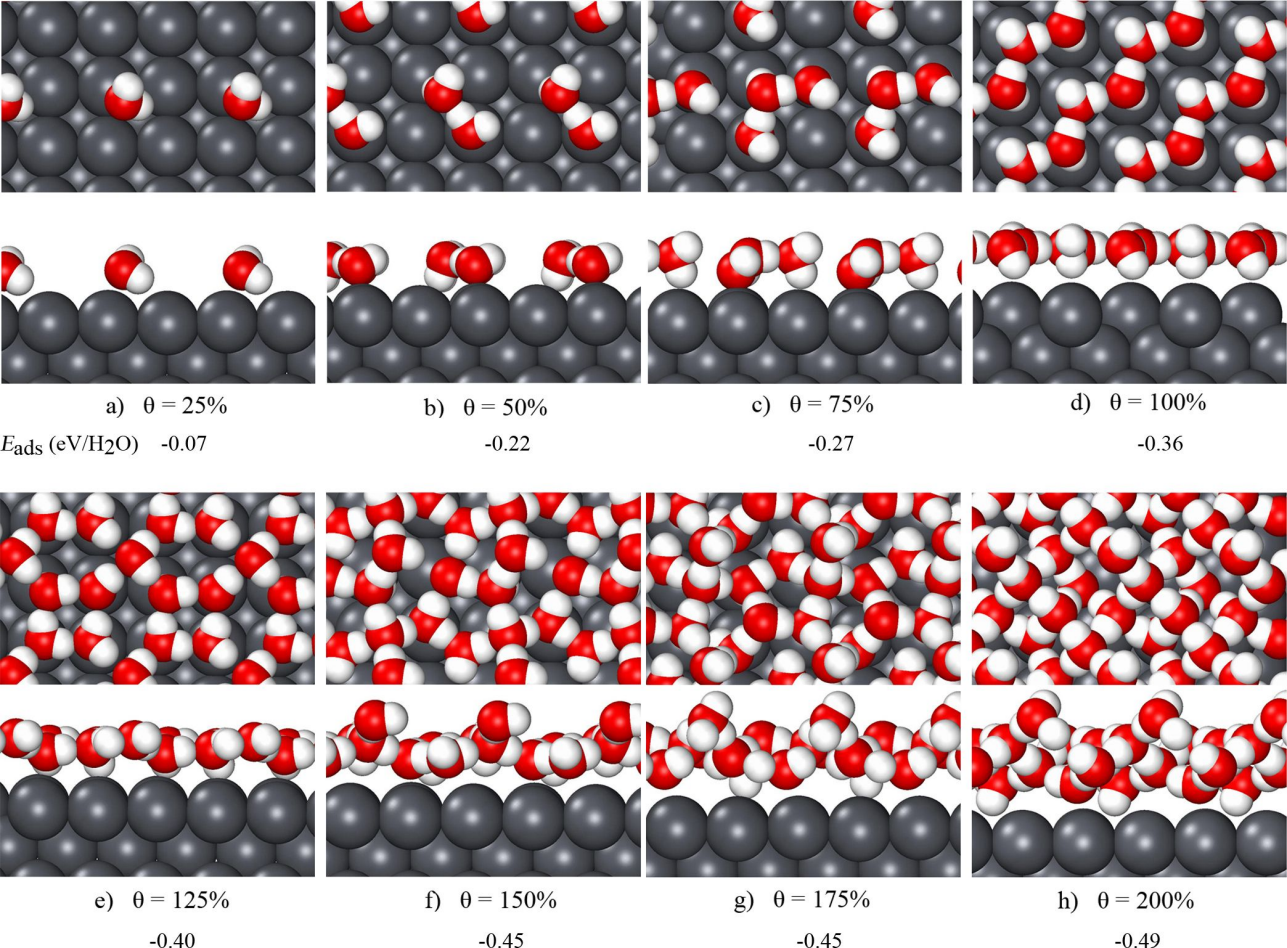
Top and side views of minimum-energy water structures on Pb(100) for coverages from 25 to 200%. The corresponding adsorption energies in electronvolts per water molecule are listed below of the corresponding panels.

Using a 2 × 2 supercell, water structures on Pb(100) with coverages ranging from 25 to 200% have been considered. The corresponding energy minimum structures are depicted in Figure 2. A single water molecule adsorbs close to a Pb top site, however, due to the already open structure of the Pb(100) the water molecule is not centered above a Pb atom, but rather canted with one hydrogen atom oriented towards the four-fold hollow site. The second water molecule adsorbs at such an hollow site as the distance between two top sites is too large to form a hydrogen bond. For the water dimer configuration shown in Figure 2b, the O–O distance amounts to 2.80 Å, and because of the additional hydrogen bond, the adsorption energy for the water dimer is about three times larger than for the water monomer. Three water molecules form a triangular structure. The water molecules roughly stay above the top sites, but the O–O distance of 2.78 Å is slightly smaller than the Pb–Pb distance. The O–O–O angle is 113°, closer to the value of 120° of a hexagonal structure than to 90° of a square structure.

These structures still do not form a connected hydrogen-bonded network. Unfortunately, to the best of our knowledge there are only few studies considering water structures on (100) metal surfaces. On non-reconstructed Au(100), DFT calculations yield a rectangular water structure at 100% coverage to be stable [16] where all water molecules are located above the ontop sites. As Figure 2d demonstrates, on Pb(100) still no closed water structure results, but rather a stripe-like structure of parallel zigzag water chains formed by an alternating arrangement of H-up and H-down water molecules. The average distance between two neighboring oxygen atoms is 2.75 Å whereas the nearest O–O distance between two neighboring water chains is 4.38 Å. This is too large for the formation of hydrogen bonds between the chains. So again, because of the large lattice constant of Pb, structural motifs that are stable on d-band metals turn out not to be stable on Pb(100).

Because of the open stripe-like structure at 100% coverage, it is reasonable that on Pb(100) a closed water layer at an even higher coverage can be obtained. This is confirmed by our DFT calculations which yield at stable water structure at 125% coverage formed by a combination of squares and distorted hexagons (see Figure 2e). One rectangle is connected to four hexagons. Compared to the layer with 100% coverage, one additional water molecule adsorbs at the hollow site between two zigzag chain. The side view in Figure 2e confirms that the water layer is rather flat and thus still truly two-dimensional. The average O–O distance is 2.98 Å, the shortest O–O bond is 2.87 Å, and the largest one 3.23 Å. Obviously, it is the water–water interaction that stabilizes this structure.

Upon adding further water molecules per unit cell, the water layer no longer stays flat, but rather a second water layer starts to form (see Figure 2f–h). Note that the adsorption energy per water molecule further increases because of the higher coordination upon formation of the second layer.

**Figure 3:**
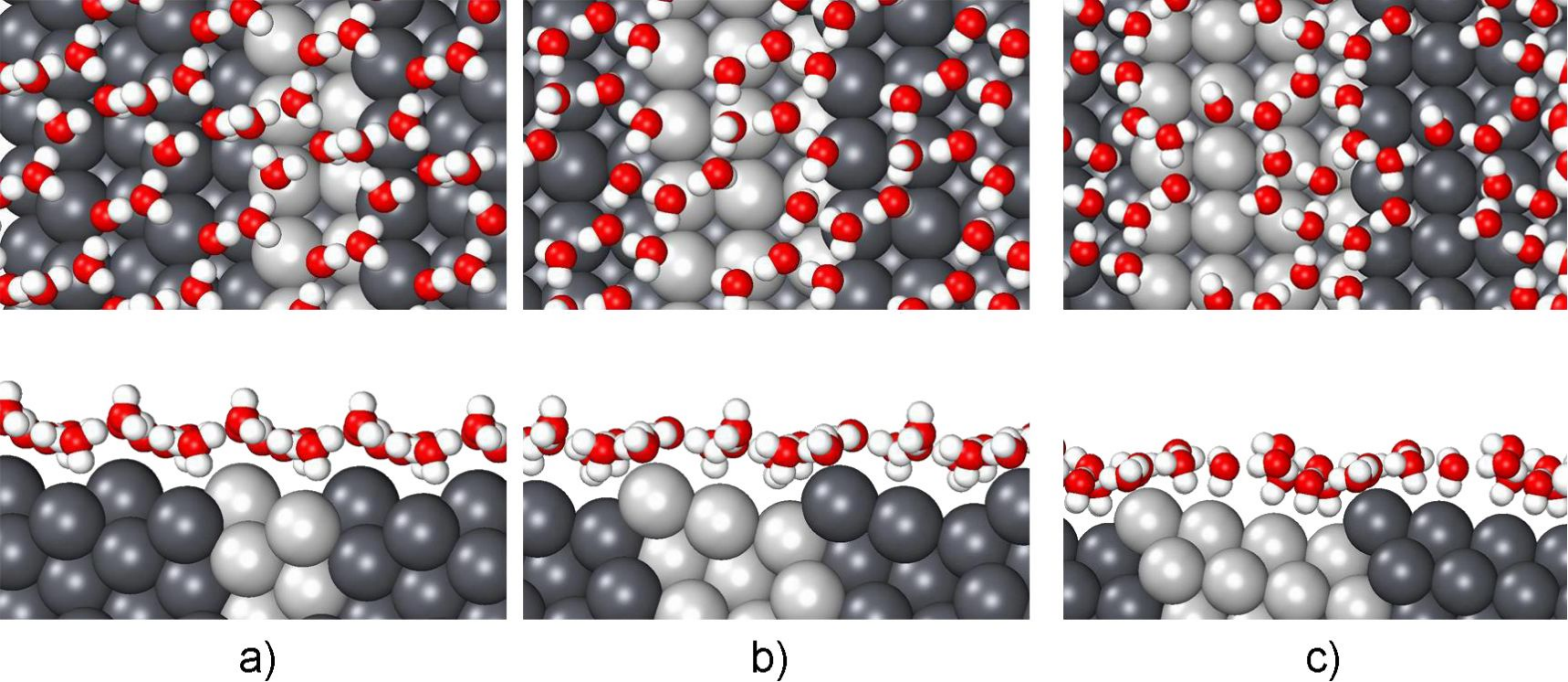
Top and side views of the energetically most stable water structures on Pb(311) (a), Pb(511) (b) and Pb(711) (c).

Next, we have considered water structures on stepped Pb(311), Pb(511) and Pb(711) surfaces. There are only few studies addressing the structure of water at stepped metal surfaces [16–17,25,57–59]. In particular, we are not aware of any studies considering the structure of water layers on stepped Pb surfaces. Hence we could not use any previous work for a guideline of our structure search. Experimentally, single water bilayers are usually prepared by a water dose of about 1 × 10^−6^ mbar resulting in a density of water molecules of about 5 × 10^14^ cm^−2^ [17,22]. Hence we used a similar density in our calculations. The stepped surfaces (*h*11) can be seen as a combination of (100) terraces with (111) steps. Therefore we used motifs of the calculated water structures on these low-index surfaces in order to obtain several different initial guesses. Interestingly enough, upon relaxation all of the structures converged to rather similar geometries indicating that our final optimized water arrangements shown in Figure 3 indeed correspond to minimum energy structures.

On Pb(311), the minimum-energy structure of water is formed by an arrangement of hexagons and rectangles which is in fact similar to the one on Pb(100). On Pb(511) and Pb(711), the resulting water structures are more complicated, apparently because of the larger terrace width, consisting of rectangles, pentagons, hexagons, including some incomplete hexagon. Note that on Au(511), the resulting water structure consists of an arrangement of distorted rectangles, hexagons and octagons [16]. However, Pb has a much larger lattice constant than Au, leading to a different water arrangement, which includes also pentagons.

In fact, five- and also seven-membered water rings have been observed in other highly packed water structures on metal surfaces [54,60–61]. However, in a recent DFT study of confined two-dimensional ice with no lateral potential variation no stable structure with seven-membered water rings has been found [62]. Also we do not obtain any seven-membered rings in our water structure optimization on the considered Pb surfaces. Apparently, this particular motif can be stabilized by a particular lateral corrugation imposed on the water layer by the substrate, but does not necessarily appear on any substrate.

Although the water structures shown in Figure 3 are different from each other, still they have common features. The water molecules at the terraces, in particular at the lower step edge, are in an H-down configuration. This allows them to form an arrangement in which all the oxygen atoms are approximately located in one plane across the step edges (see the side views in Figure 3), similar to what has been found for water on Au(511) [16].

Note that the adsorption energy of single water molecules on flat Pb surfaces is rather small, it only amounts to −0.07 eV both on Pb(111) and Pb(100)). However, at the step edge of the stepped Pb surfaces a water monomer binds much stronger to Pb, reflected in adsorption energies of −0.20, −0.39 and −0.39 eV on Pb(311), Pb(511) and Pb(711)), respectively. As a consequence, the water layer is pinned at the Pb step atoms where the water molecules above the terraces are farther away from the Pb atoms. On Pb(711) for example, the average distance between the oxygen atoms of the water molecule above the terrace and the Pb surface atoms is 4.74 Å. This is indicative of a relatively weak metal–water interaction above the terraces, which will on the other hand make the water–water interaction stronger [15].

**Figure 4:**
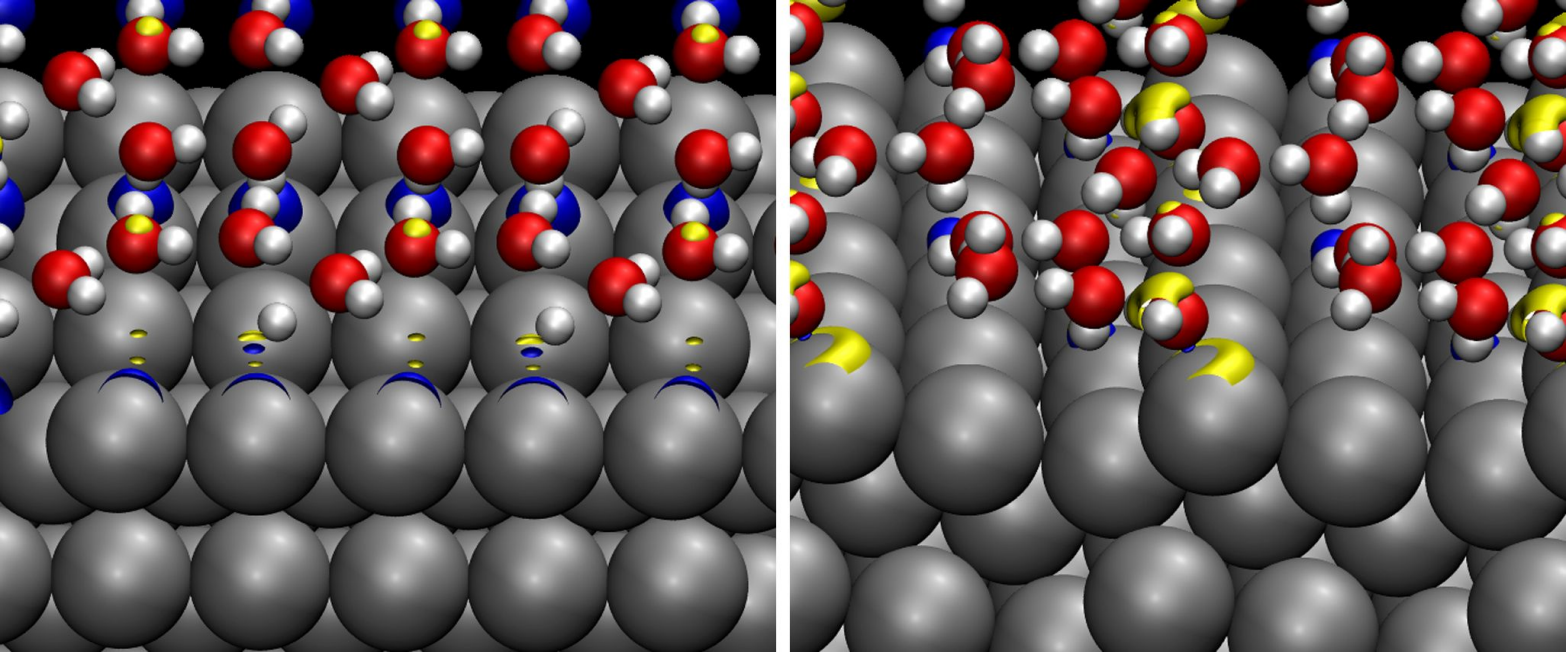
Isosurface plots of charge-density differences upon water adsorption on a) Pb(100) and b) Pb(511). The plotted isosurfaces correspond to charge density with an absolute value of 0.006 *e*/Å^2^. Charge accumulation, i.e., an increase in the electron density, is plotted in yellow, charge depletion in blue.

In order to understand the differences between the water adsorption on the terraces and the steps, we have determined the charge density difference upon water adsorption on Pb(100) and Pb(511) (Figure 4) which corresponds to the adsorption-induced charge rearrangement. On Pb(100), there is a charge accumulation below the water molecules which bind through their oxygen atom to the Pb atoms and a charge depletion below the water molecule in the H-down configuration. However, the polarization of the adsorbed water molecules on Pb(100) is significantly smaller than for example on Au [16] which reflects that there is only a rather weak interaction between Pb and water.

On the stepped Pb(511) surface, the charge rearrangement is mainly localized at the step edge, similar to the water/Au(511) system [16]. The Pb atoms at the terrace sites do not exhibit any significant charge rearrangement upon the formation of the water layer. This confirms the general picture of water adsorption at stepped surfaces [15]: Water tends to form a flat layer that rests on the step atoms, but above the terraces it almost corresponds to a free-standing layer.

**Figure 5:**
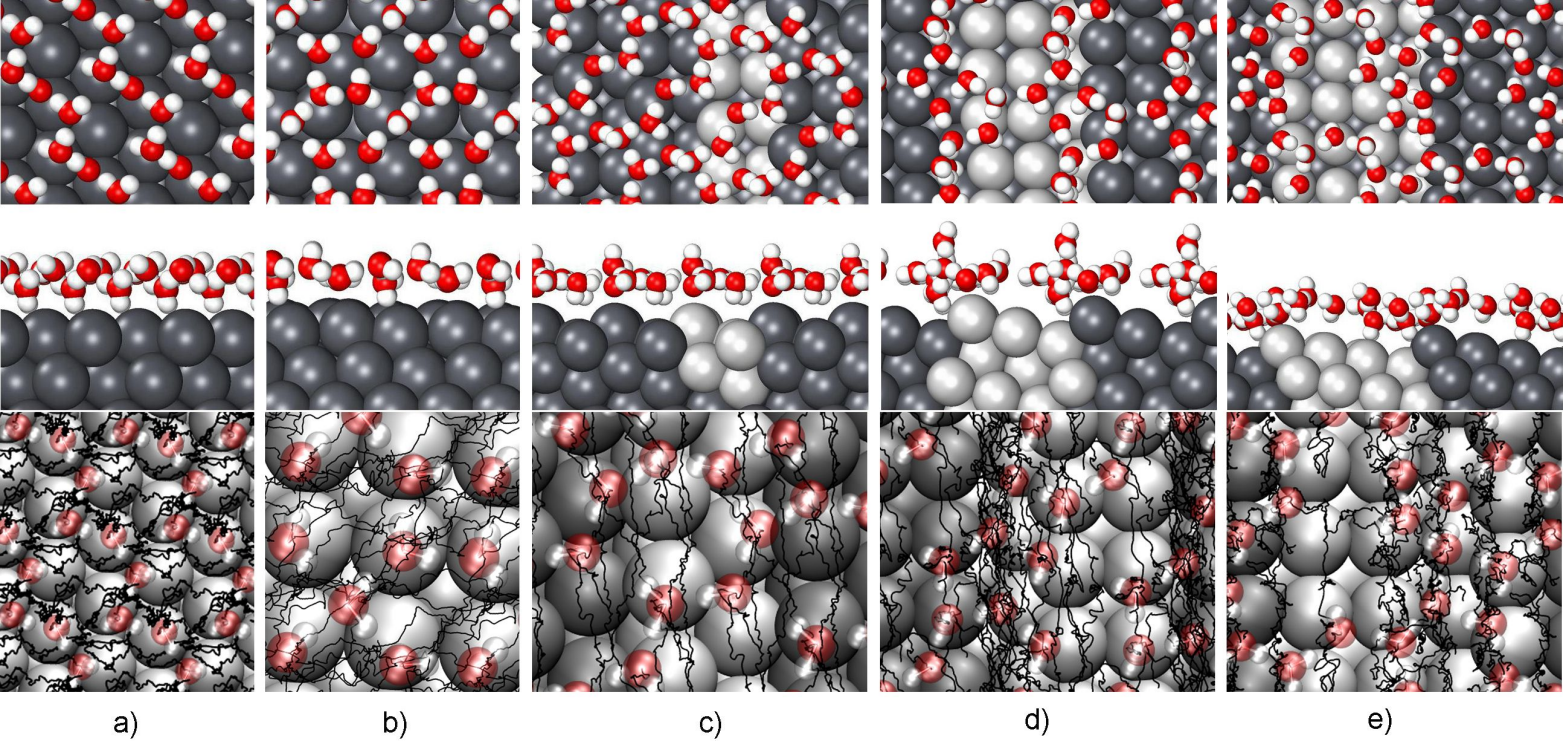
Upper two panels: Snapshots of the AIMD simulation of water layers at a temperature of 140 K on a) Pb(111), b) Pb(100), c) Pb(311) d) Pb(511) and e) Pb(711) in top and side view; lower panel: trajectories of the oxygen atoms of the water molecules along the AIMD run indicated by black lines. For Pb(111) and Pb(100), the water coverages correspond to the most stable flat water structures, i.e., 1 and 5/4, respectively.

The thermal stability of the water structures has been checked by performing AIMD simulations at a temperature of 140 K for a run time of 8 ps. This temperature has been chosen as it is slightly below the typical desorption temperature of single water layers on metal substrates [22]. The resulting structure and trajectories are illustrated in Figure 5. For Pb(111) and Pb(100), the most stable flat water structures illustrated in Figure 1c and Figure 2e have been chosen as initial conditions, for the stepped Pb surfaces the corresponding structures shown in Figure 3.

On Pb(111), the water structure in fact starts to dissolve within the run time of 8 ps at 140 K. Figure 5a shows that there is no longer a flat water layer, but a kind of bilayer with the distance between the O atom of the upper water molecules and the Pb surface being 5.2 Å, which is 2.4 Å larger than for the lower water molecules. The plotted trajectories demonstrate that the water molecules leave their initial positions as a consequence of the weak Pb–water interaction.

On the other high-index surface, Pb(100), the water layer exhibits a higher stability than on Pb(111). There is almost no indication of any structural rearrangement during the simulation time. Upon thermalization, the average oxygen-surface distance only increases from 3.79 Å to 4.02 Å, the height difference between the upper most and lowest water molecules is 0.80 Å (= 4.26 Å − 3.46 Å). Still, the plotted trajectories demonstrate that the water molecules are not fixed but move along the surface. However, the water molecules do not move independently but rather as a whole keeping the water arrangements. This indicates a relatively strong water–water interaction within the network consisting of rectangles and distorted hexagons. The corresponding water–water interaction in the free-standing configuration amounts to 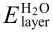 = −0.37 eV, which is significantly stronger than water–water interaction in the Pb(111) geometry reflected by 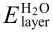 = −0.22 eV (see Table 1). Obviously, the strong water–water interaction on Pb(100) keeps the water layer intact. On the other hand, the water–Pb interaction is rather weak so that the layer can move relatively freely along the surface.

As far as the water structures on stepped surfaces at 140 K are concerned, on Pb(311) and Pb(711) the water layers are also rather stable (see Figure 5c,e), similarly to what has been observed in AIMD simulations of a water layer on Au(511) [16]. On one hand, the water layers are pinned to the Pb step edge atoms because of the strong water–metal interaction there, on the other hand, the water molecules above the terrace form a stiff hydrogen-bonded network as they are hardly interacting with the underlying Pb atoms. Still, the trajectories depicted in the lower panels of Figure 5c,e show that the water molecules move on Pb(311) and Pb(711) at a temperature of 140 K, but only in the direction parallel to the steps. So the pinning of the water molecules at the step edge prevents the water structure from being shifted away from the steps, but not along the steps.

However, this mechanism does not seem to be operative for the water layer on Pb(511), as Figure 5d demonstrates. At the end of the 8 ps AIMD run, one water molecule has left the first water layer and starts the formation of a second layer. Still, again the water molecules mainly move parallel to the steps and not perpendicular to them. It might be that the particular width of the terraces of the Pb(511) surfaces does not favor the formation of a stable hydrogen-bonded water network.

**Figure 6:**
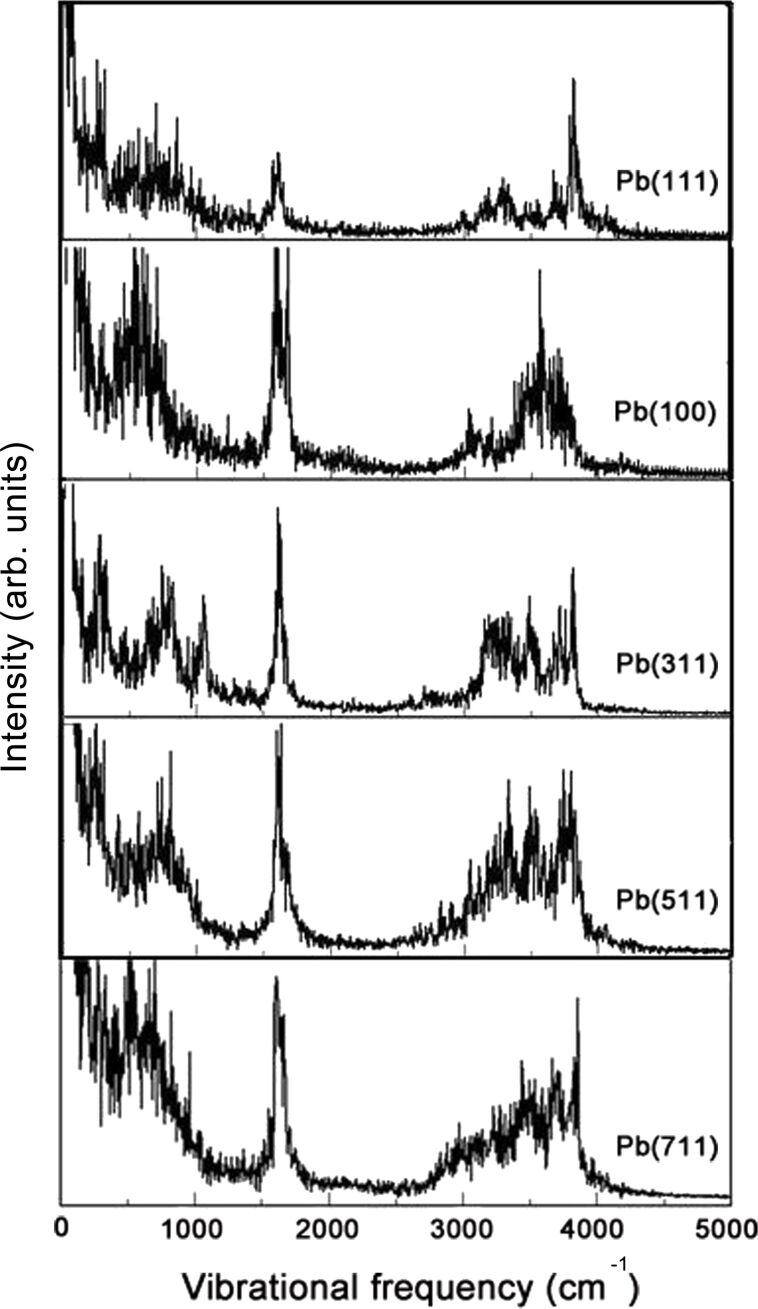
Vibrational spectra of the water layer on Pb(111), Pb(100), Pb(311), Pb(511) and Pb(711) derived from the Fourier transform of the velocity auto-correlation function.

On silver and gold surfaces, vibrational spectra of water layers on stepped surfaces have been determined both experimentally [17,25] and numerically [16]. Using the Fourier transform of the velocity auto-correlation function, we also derived the vibrational spectrum of the water layers on the considered Pb surfaces at the temperature of 140 K (see Figure 6). These spectra have been calculated by averaging over five different initial configurations in the determination of the velocity auto-correlation function. As dipole selection rules have not been taken into account in the derivation of the spectra, they can not be quantitatively compared to infrared (IR) spectra as far as the intensity of the peaks is concerned, the positions of the peaks, however, are not influenced by this.

On all considered surfaces, a sharp peak in the vibrational spectra shown in Figure 6 is visible at about 1600 cm^−1^. This peak is related to the hydrogen scissor mode in the water molecules and has also been clearly observed in the experiments of water on stepped Au and Ag surfaces [17,25]. In the O–H stretching region above 3000 cm^−1^, on the low-index Pb surfaces there is only one prominent peak at about 3700 cm^−1^ on Pb(111) and 3600 cm^−1^ on Pb(100).

On the stepped surfaces, this peak is split into three modes, two stretching modes at 3200 cm^−1^ and 3500 cm^−1^ which are related to H-bonded (HB) hydrogen, and a further mode at 3700 cm^−1^ that has been attributed to non-H-bonded (NHB) hydrogen [17]. Again, similar observations have been made on Au and Ag surfaces [17,25]. Interestingly enough, although the water structure on Pb(311) is rather similar to the one on Pb(100), the spectra are different. Furthermore, the splitting into the three stretch modes becomes less obvious with increasing terrace width. This can be explained by the fact that with increasing terrace width the step contribution becomes less dominant so that the spectra should become more similar to the one on Pb(100).

## Conclusion

The structural, electronic and vibrational properties of water layers on flat and stepped Pb surfaces have been studied using density functional theory calculations. On Pb(111) and Pb(100), the energetically most favorable water structures differ from those on the corresponding transition metal surfaces because of the much larger lattice constant of Pb. On the stepped surfaces, water forms flat layers that are pinned to the Pb step atoms but very weakly interacting with the terrace atoms, as confirmed by an analysis of the charge density difference upon water adsorption.

The thermal stability of the water layers has been addressed by performing ab initio molecular dynamics simulations at a temperature of 140 K. The minimum-energy structure of the water layers on Pb(111) and Pb(511) turned out to be unstable at this temperature. In contrast, on Pb(100), Pb(311) and Pb(711) the water layers remained intact during the run time of the AIMD simulations of 8 ps because of the stronger hydrogen-bonded network. Still, because of the relatively weak Pb–water interaction, the water layers can easily move along the surface as a whole.

The AIMD simulations have also been used to derive vibrational spectra of the adsorbed water layers. On the low-index Pb surfaces, only one peak has been observed in the O–H stretching region, whereas there is a splitting of this mode into three peaks at about 3200 cm^−1^, 3500 cm^−1^ and 3700 cm^−1^. The two peaks at the lower wavenumbers are assigned to H-bonded hydrogen whereas the peak with the highest wavenumber is attributed to non-H-bonded hydrogen. The properties of the water layers on the stepped Pb surfaces are similiar to those found on stepped Ag and Au surfaces.

Because of the weak Pb–water interaction apparent in the calculations it should be expected that the presence of water only has a weak influence of the microscopic mechanism leading to the formation of the atomic switch. The same should be true as far as the electronic transport properties of the switch are concerned.

## Acknowledgements

Financial support by the Baden-Württemberg Foundation through the project C3 within the network “Functional Nanostructures” is gratefully acknowledged. Computational resources have been provided by the bwHPC5 project of the Federal State of Baden-Württemberg, Germany.
